# Supplementary material for: Structural and mechanistic basis of differentiated inhibitors of the acute pancreatitis target kynurenine-3-monooxygenase
Source: Nat Commun. 2017 Jun 12;8:15827. doi: 10.1038/ncomms15827 (PMC5477544; doi:10.1038/ncomms15827)
Supplement: Supplementary Information — Supplementary Figures, Supplementary Tables and Supplementary References. [file ncomms15827-s1.pdf]

SUPPLEMENTARY FIGURES

KMO protein sequence alignment (Boxed residues are within **4Å** of UPF648 and are all conserved except Tyr 323)

```
1    MDSSVIQRKKVAVIGGGLVGSLQACFLAKRNFQIDVYEAREDTRVATFTRG--RSINLALSHRGRQAL Human_O15229
1    ------MSESVAIIGAGLVGCLAALAFSKEGYNVTLYDFRQDPRLDTTKNKNLKSINLAISARGIDAL Saccharomyces_P38169
1    -MTATDNARQVTIIGAGLAGTLVARLLARNGWQVNLFERRPDPRIETGARG--RSINLALAERGAHAL Pseudomonas_Q84HF5
                                                              53

67   KAVG--LEDQIVSQGIPMRARMIHSLSGKKSAIPYG-TKSQYILSVSRENLNKDLLTAAEKYPNVKMH Human_O15229
63   KSIDPDACEHILQDMIPMKGRMIHDLKGRQESQLYG-LHGEAINSINRSVLNNSLLDELEKS-TTELK Saccharomyces_P38169
66   RLAG--LEREVLAEAVMMRGRMVHVPGTPPNLQPYGRDDSEVIWSINRDRLNRILLDGAEAA-GASIH Pseudomonas_Q84HF5
                         83             97     104

132  FNHRLLKCNPEEG---MITVLGSD-KVPKDVTCDLIVGCDGAYSTVRSHLMKKPRFDYSQQYIPHGYM Human_O15229
129  FGHKLVKIEWTDDKQICHFAIGEDLKTPHTEKYDFVIGCDGAYSATRSQMQRKVEMDFSQEYMNLRYI Saccharomyces_P38169
131  FNLGLDSVDFARQRLTLSNVSGER----LEKRFHLLIGADGCNSAVRQAMASVVDLGEHLETQPHGYK Pseudomonas_Q84HF5
                                                169

196  ELTIPP-------KNGDYAMEPNYLHIWPRNTFMMIALPNMNKSFTCTLFMPFEE---------FEKL Human_O15229
197  ELYIPPTEEFKPNYGGNFAIAPDHLHIWPRHKFMLIALANSDGSFTSTFFGSKDQ---------ISDL Saccharomyces_P38169
195  ELQITP------EASAQFNLEPNALHIWPHGDYMCIALPNLDRSFTVTLFLHHQSPAAQPASPCFAQL Pseudomonas_Q84HF5
                             221     230 232        246

248  LTSN-DVVDFFQKYFPDAIPLIGEKLLVQDFFLLPAQPMISVKCSSFHFKS-HCVLLGDAAHAIVPFF Human_O15229
256  ITSKSRVREFLIENFPDIINIMDLDDAVKRFITYPKESLVCVNCKPYDVPGGKAILLGDAAHAMVPFY Saccharomyces_P38169
257  VDGH-AARRFFQRQFPDLSPMLDS--LEQDFEHHPTGKLATLRLTTWHVGG-QAVLLGDAAHPMVPFH Pseudomonas_Q84HF5
                                                                321/322/323

314  GQGMNAGFEDCLVFDELMDKFSNDLSLCLPVFSRLRIPDDHAISDLSMYNYIEMRAHVNSSWFIFQKN Human_O15229
324  GQGMNCGFEDVRILMALLKKHSGDRSRAFTEYTQTRHKDLVSITELAKRNYKEMSHDVTSKRFLLRKK Saccharomyces_P38169
321  GQGMNCALEDAVALAEHLQSAADNAS-ALAAFTAQRQPDALAIQAMALENYVEMSSKVASPTYLLERE Pseudomonas_Q84HF5
324

382  MERFLHAIMPSTFIPLYTMVTF-SRIRYHEAVQRWHWQKKVINKGLFFLGSLIAISSTYLLIHYMSPR Human_O15229
392  LDALFSIIMKDKWIPLYTMISFRSDISYSRALERAGKQTRILKFLESLTLGMLSIGGYKLFKFLTRER Saccharomyces_P38169
388  LGQIMAQRQPTRFIPRYSMVTF-SRLPYAQAMARGQIQEQLLKFAVANHSDLTSINLDAVEHEVTRCL Pseudomonas_Q84HF5
        394

449  SFLRLRRPWNWIAHFRNTTCFPAKAVDSLEQISNLISR                               Human_O15229
460  S                                                                    Saccharomyces_P38169
455  PPLSHLC                                                              Pseudomonas_Q84HF5
```

Residues deleted in yeast crystal structure construct

Sequence identities: human/yeast 38%, human/pseudomonas 35%, yeast/pseudomonas 35%

Supplementary Figure 1 Alignment of KMO sequences of Human, Scacharmyces and Pseudomonas fluorescens

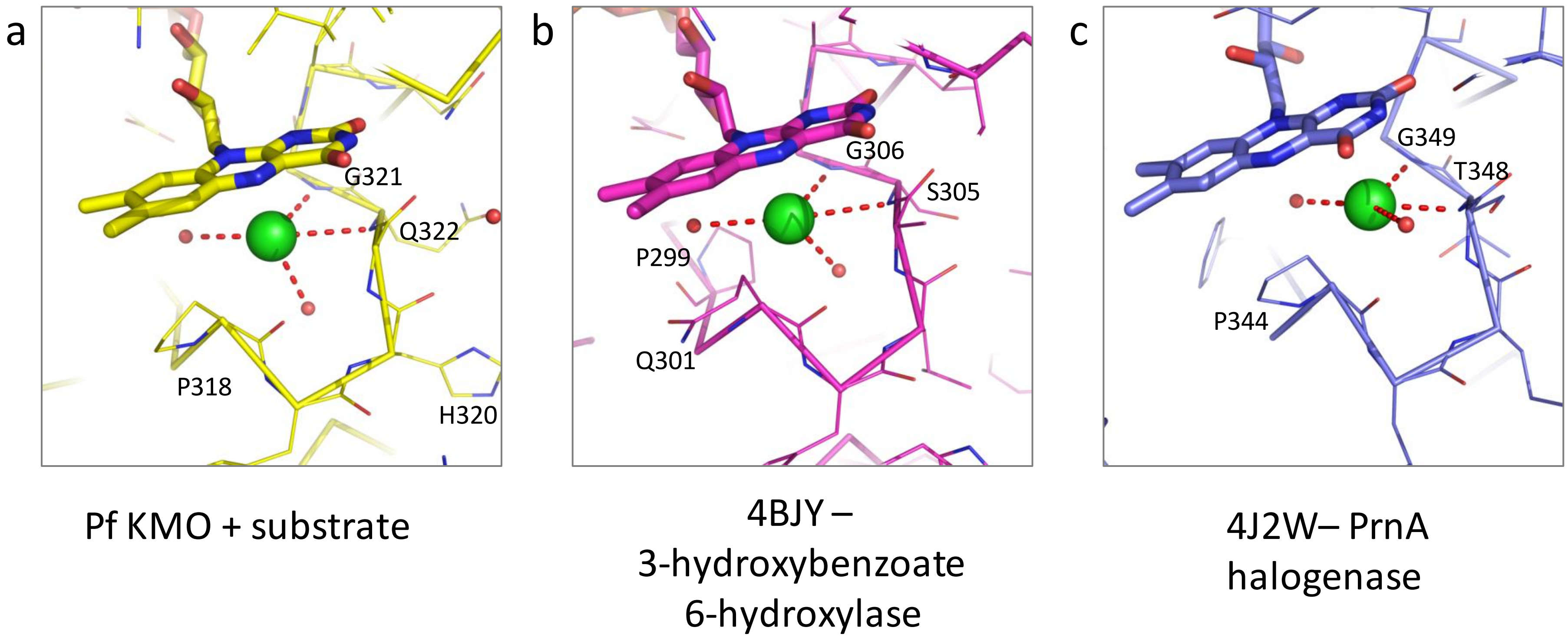

Supplementary Figure 2 Similarity of chloride ion promixal to catalytic flavin of three FAD-dependent enzymes
(a) Pf-KMO, **(b)** 3-hydroxybenzoate6-hydroxylase (PDB: 4BJY) and **(c)** PrnA halogenase (PDB: 4J2W)

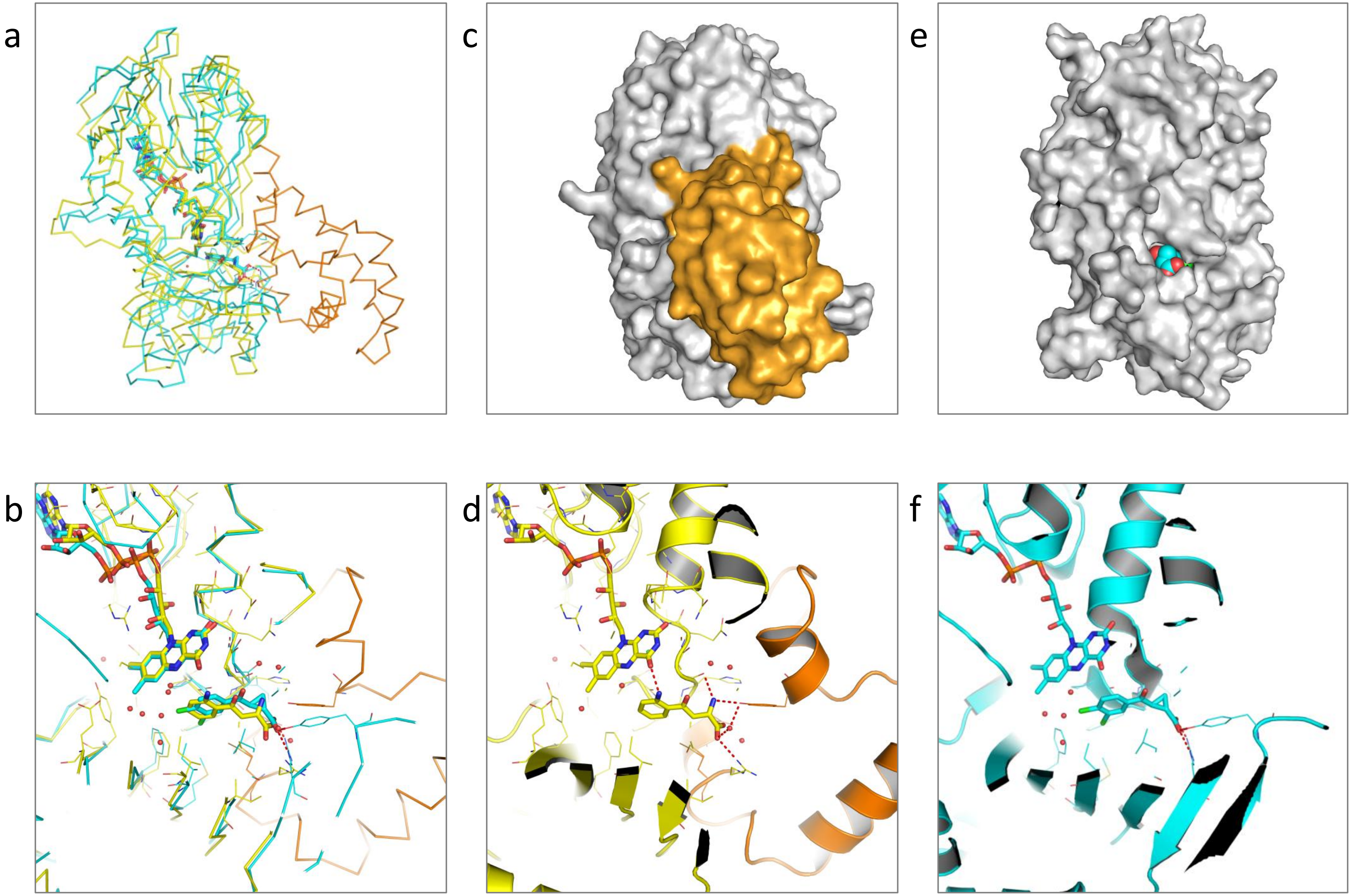

Supplementary Figure 3 Comparison of Pf-KMO-Kyn substrate complex with Saccharomyces cerevisiae KMO-394UPF inhibitor complex (cyan carbons, PDB 4J36)
(a) Structural overlay of yeast (cyan) and Pf structures (domain1 and 2 in yellow, domain 3 in orange)). Domain 3 is not present in the protein crystallised yeast structure. **(b)** Enlarged view of (a) focused on the active site. **(c)** Pf-KMO-Kyn substrate complex shown as surface representation with orange colouring to highlight domain 3 **(d)** Enlarged view of the Pf-KMO active site of (c) without protein surface **(e)** Yeast structure in the same orientation as (b) highlighting the substrate capping effect of domain 3 **(f)** Enlarged view of the active site of Saccharomyces cerevisiae (c) without protein surface.

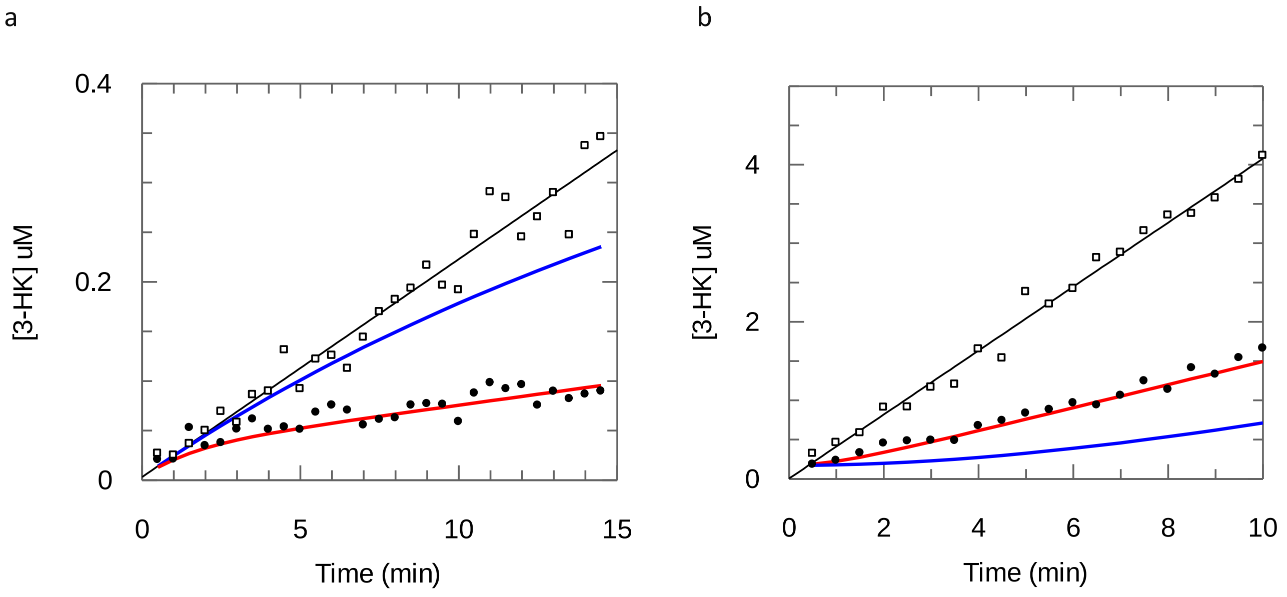

Supplementary Figure 4. Simulations of association and dissociation timecourses for inhibition of KMO by GSK428(A2)

(a) Onset of inhibition for GSK428(A2). The uninhibited timecourse (open symbols) was fitted to a straight line to give the uninhibited rate (V_i = 0.022 μM/min), the gradient of the last 5 minutes of the timecourse in the presence of 10 nM GSK428(A2) (closed symbols) was taken as the steady-state rate (V_s = 0.004 μM/min). These two values were used to simulate timecourses for onset of inhibition with k_{obs} = 0.062 min^{-1} (blue) and k_{obs} = 0.62 min^{-1} (red), using Equation 1 (Supplementary Methods). **(b)** Dissociation of GSK428(A2). The uninhibited timecourse (open symbols) was fitted to a straight line to give the uninhibited rate, the gradient of the last 5 minutes of the timecourse in the presence of GSK428(A2) (closed symbols) was taken as the steady-state rate after equilibration of inhibitor binding (V_s = 0.147 μM /min). This value was used to simulate timecourses for recovery of activity with k_{obs} = 0.1 min^{-1} (blue) and k_{obs} = 1.0 min^{-1} (red), using Equation 1 with V_i set to zero.

a

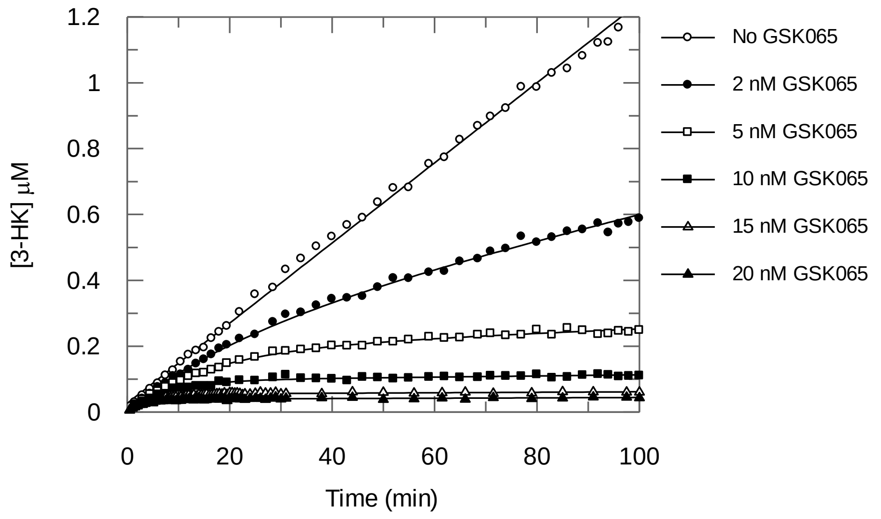

b

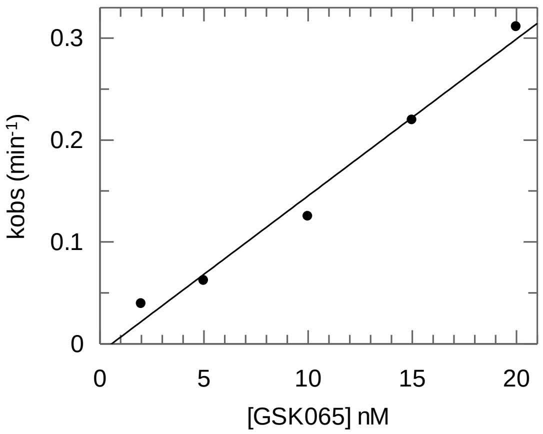

c

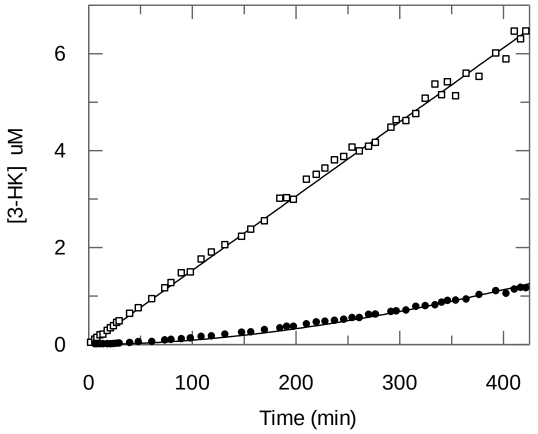

Supplementary Figure 5. Detailed kinetic analysis of inhibition of KMO by GSK065(C1)

(a) Onset of inhibition timecourses measured in a range of concentrations of GSK065(C1). k_{obs} at each inhibitor concentration was obtained by fitting timecourses to Equation 1 (Supplementary Methods). **(b)** Plot of the resulting k_{obs} values against [GSK065(C1)]; k_{on} was obtained from a linear fit of the data. k_{on} = 6.3 x 10^5 $M^{-1}s^{-1}$ (average of two determinations). **(c)** Dissociation of GSK065(C1) from KMO. Recovery of activity timecourse was measured after adding a high concentration of L-Kyn to a pre-equilibrated mixture of KMO and inhibitor (closed symbols). [GSK065(C1)] = 5 nM after addition of L-Kyn. The uninhibited timecourse was generated in an equivalent way, omitting the inhibitor (open symbols). k_{off} for the inhibitor was obtained by simultanously fitting both timecourses to a competitive binding model (Supplementary Methods), with k_{on} fixed at 6.3 x 10^5 $M^{-1}s^{-1}$ (see above). k_{off} = 3.2 x 10^{-5} s^{-1} (average of two determinations).

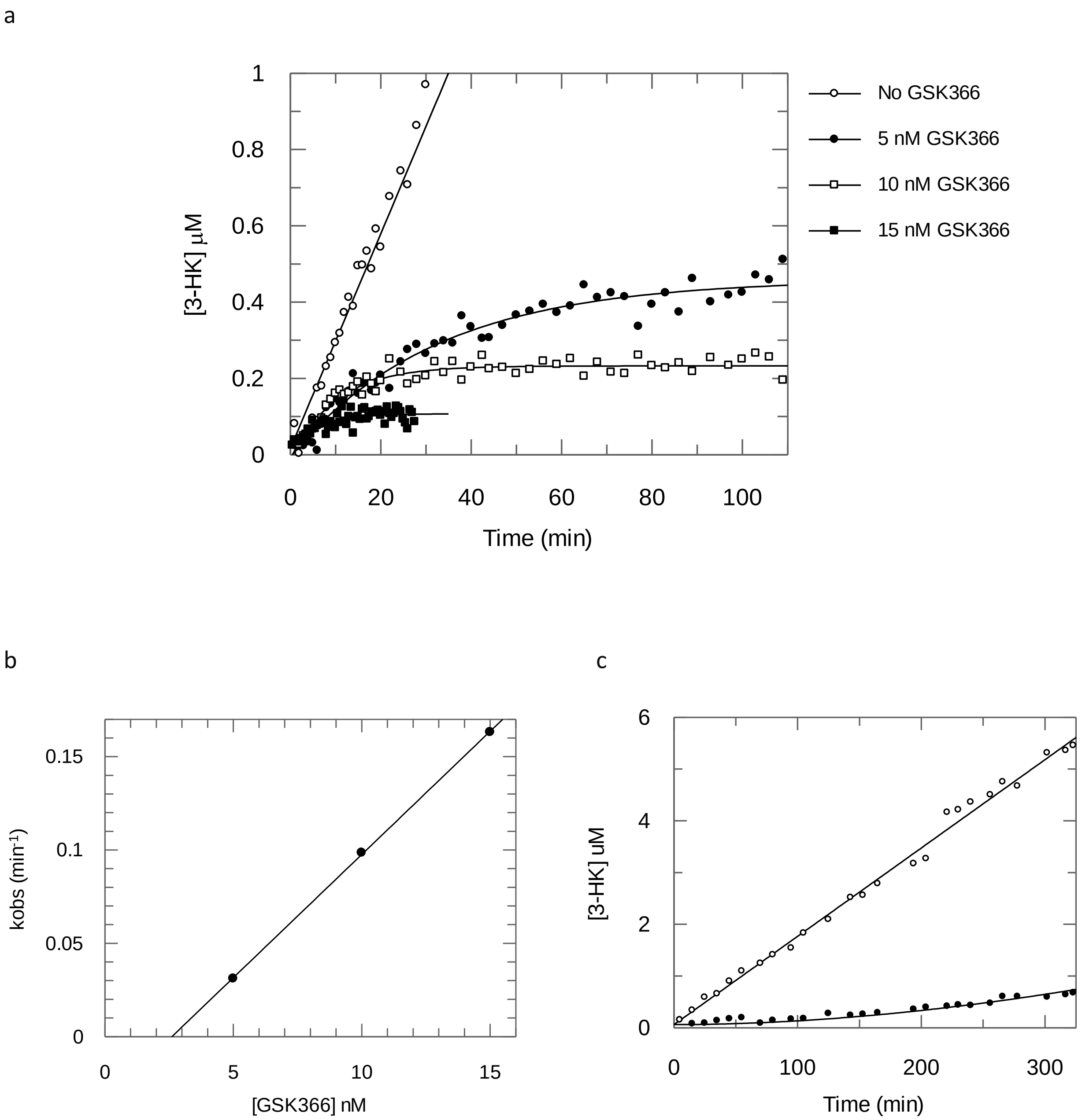

Supplementary Figure 6. Detailed kinetic analysis of inhibition of KMO by GSK366(C2)

(a) Onset of inhibition timecourses measured in a range of concentrations of GSK366(C2). k_{obs} at each inhibitor concentration was obtained by fitting timecourses to Equation 1 (Supplementary Methods). **(b)** Plot of the resulting k_{obs} values against [GSK366(C2)]; k_{on} = 1.3 x 10^6 $M^{-1}s^{-1}$ was obtained from a linear fit of the data. **(c)** Dissociation of GSK366(C2) from KMO. A recovery of activity timecourse was measured after adding a high concentration of L-Kyn to a pre-equilibrated mixture of KMO and inhibitor (closed symbols). [GSK366(C2)] = 5 nM after addition of L-Kyn. The uninhibited timecourse was generated in an equivalent way, omitting the inhibitor (open symbols). k_{off} for the inhibitor was obtained by simultanously fitting both timecourses to a competitive binding model (Supplementary Methods), with k_{on} fixed at 1.3 x 10^6 $M^{-1}s^{-1}$ (see above). k_{off} = 1.6 x 10^{-5} s^{-1} (average of two determinations).

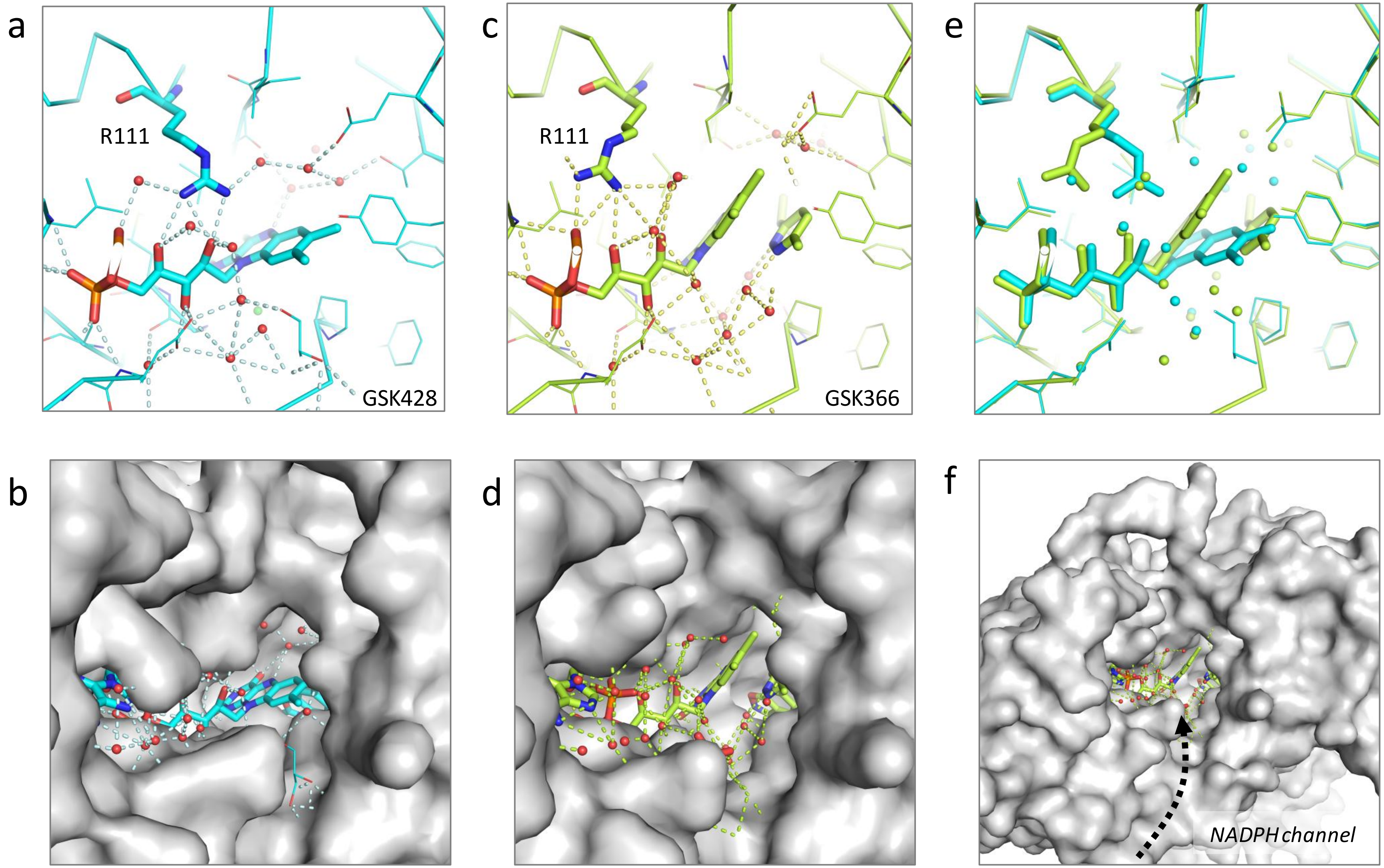

Supplementary Figure 7. Comparison of Pf-KMO complexes of GSK428 (cyan) and GSK366(C2) (pale green) viewed from solvent channel and putative NADPH access site

(a-b) Enclosed active site of GSK428(A2) with flavin in *in* position, substrate is not visible from solvent channel **(c-d)** Same views as (a-b) but using GSK366(C2) complex **(e-f)** Overlay of structures of GSK428(A2) (cyan) and GSK366(C2)(pale green). Movement of R111 visible.

L-kynurenine complex

A subunit

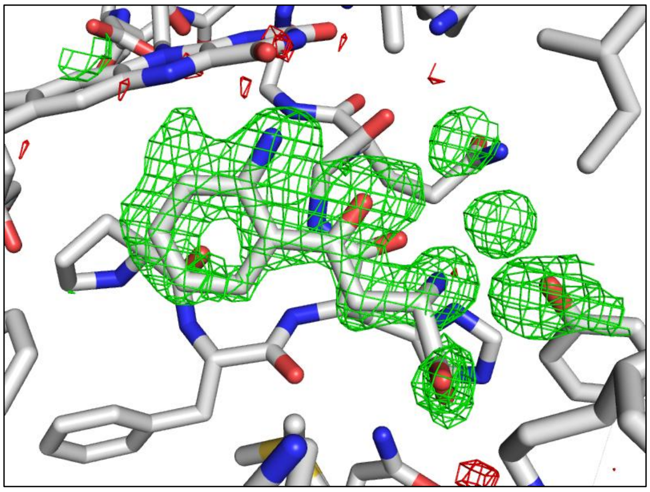

B subunit (ligand not bound)

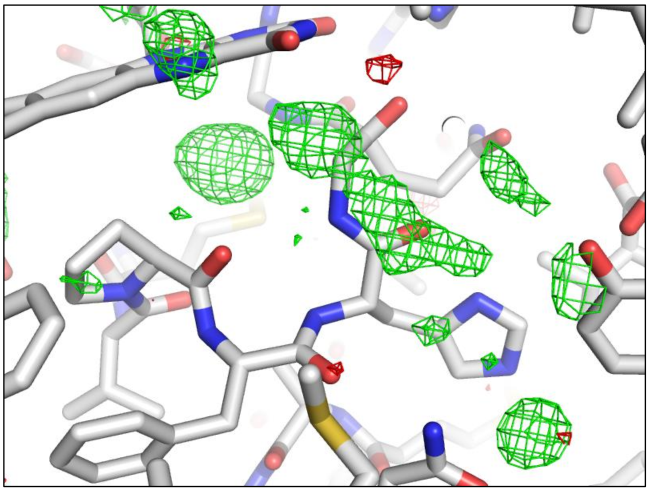

GSK428(A2) complex

A subunit

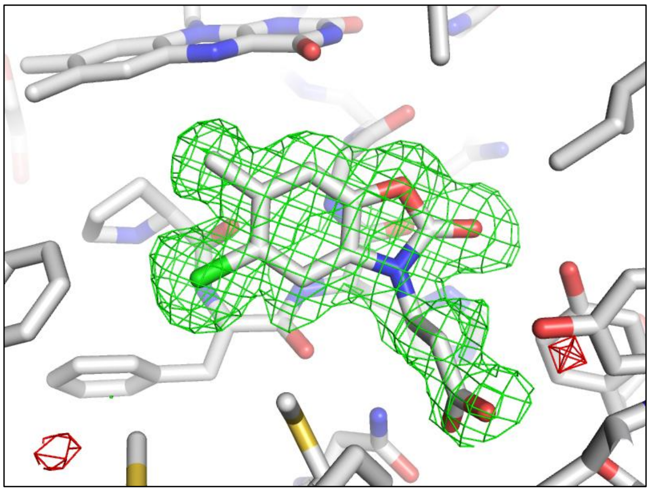

B subunit

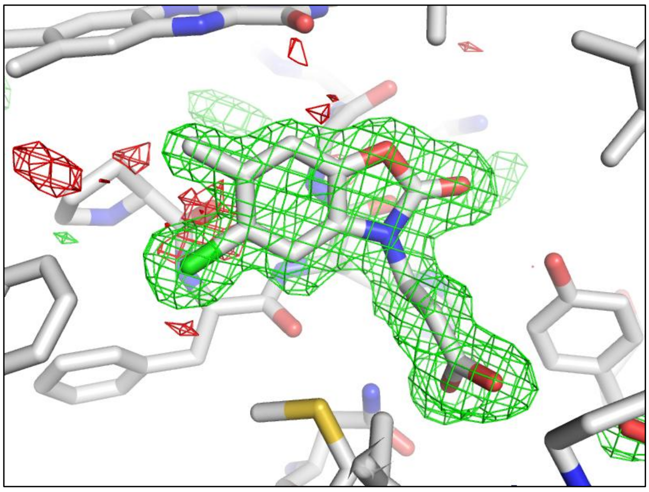

Supplementary Figure 8. Electron density difference maps in the inhibitor binding sites.

Initial F_o-F_c electron density maps contoured at 3σ (green) and -3σ (red) superposed on the refined protein-inhibitor complex structures. Maps calculated after preliminary refinement of an unliganded protein model against the structure factors of the protein-inhibitor complex.

GSK775(B2) complex

A subunit

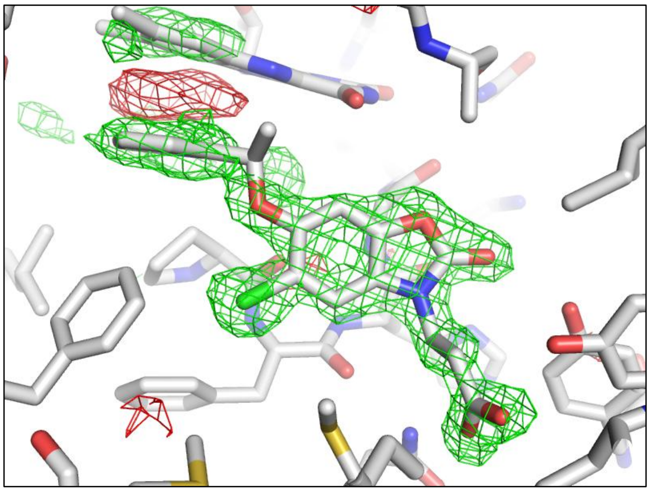

B subunit

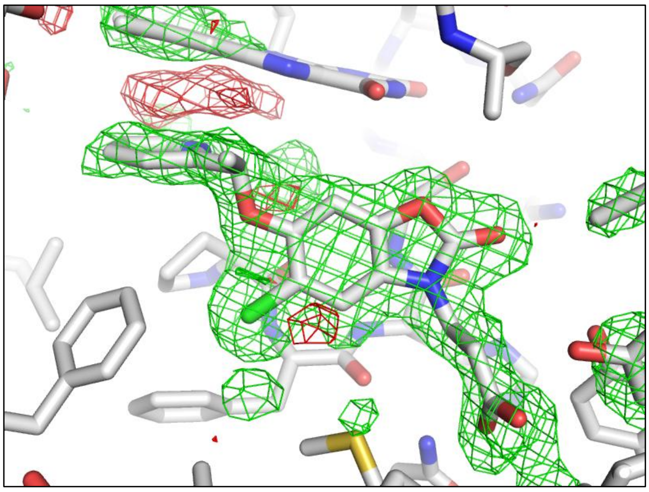

GSK065(C1) complex

A subunit

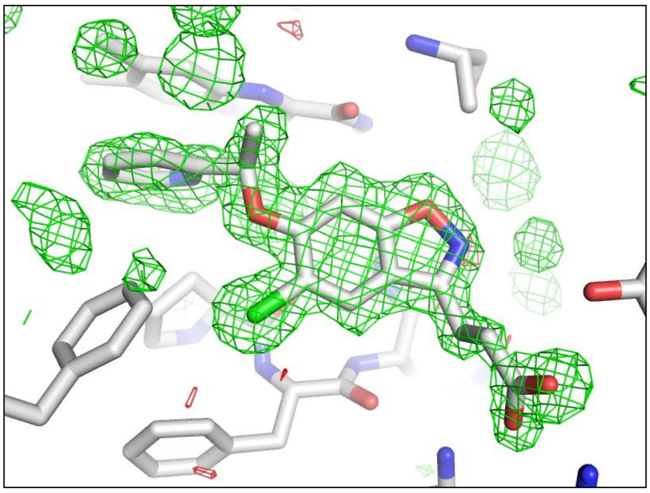

B subunit

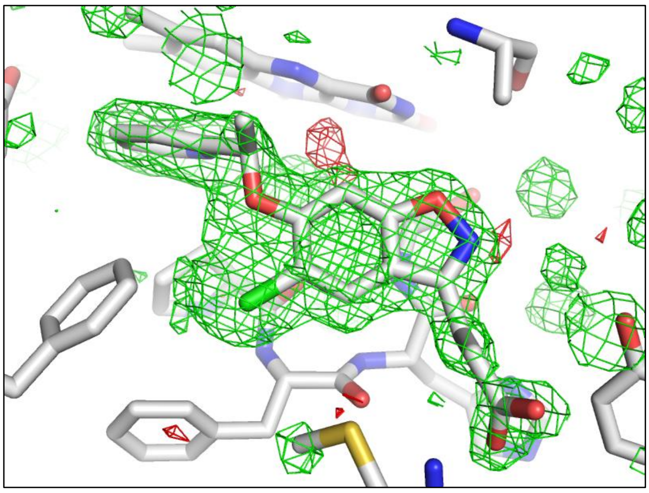

GSK366(C2) complex

A subunit

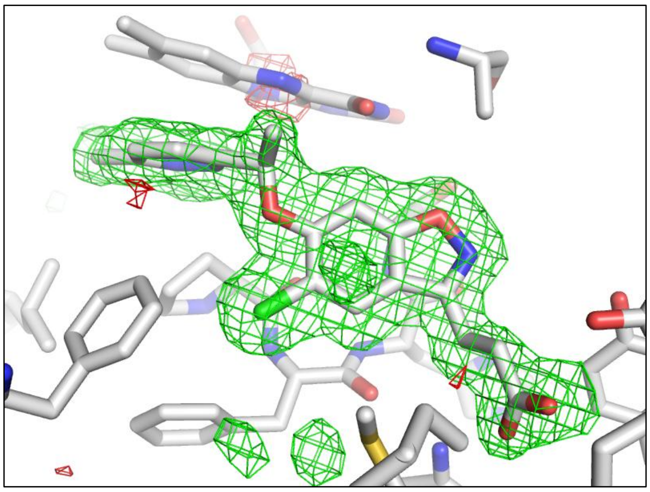

B subunit

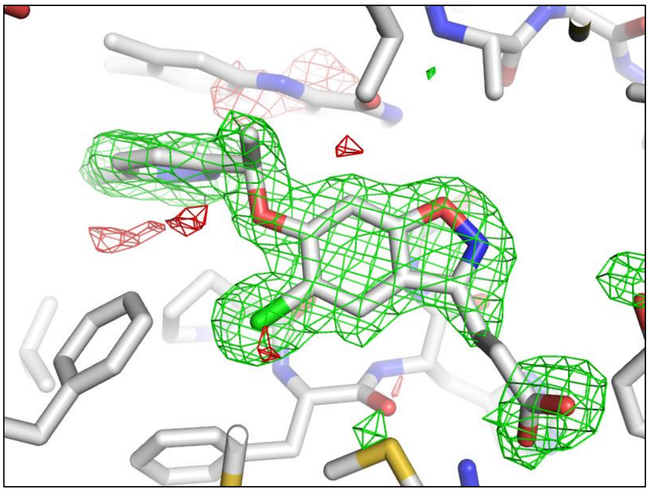

Supplementary Figure 8 cont. Electron density difference maps in inhibitor binding sites.

L-kynurenine complex (A subunit)

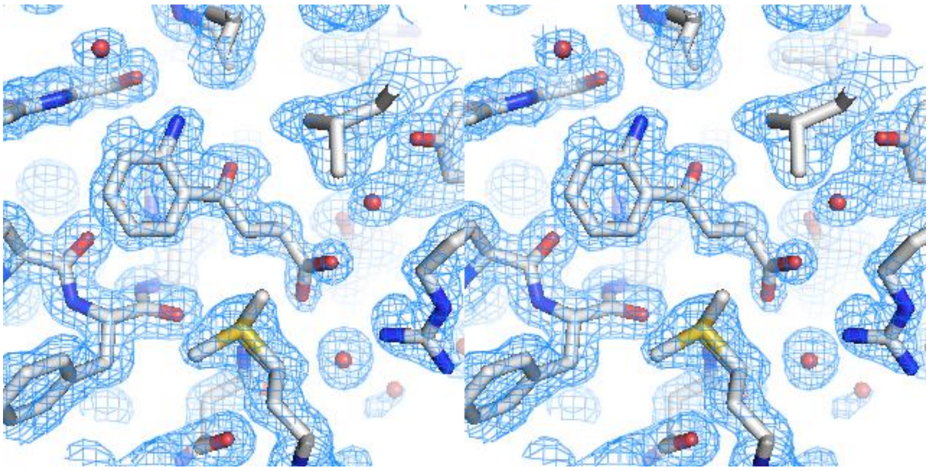

GSK428(A2) complex (A subunit)

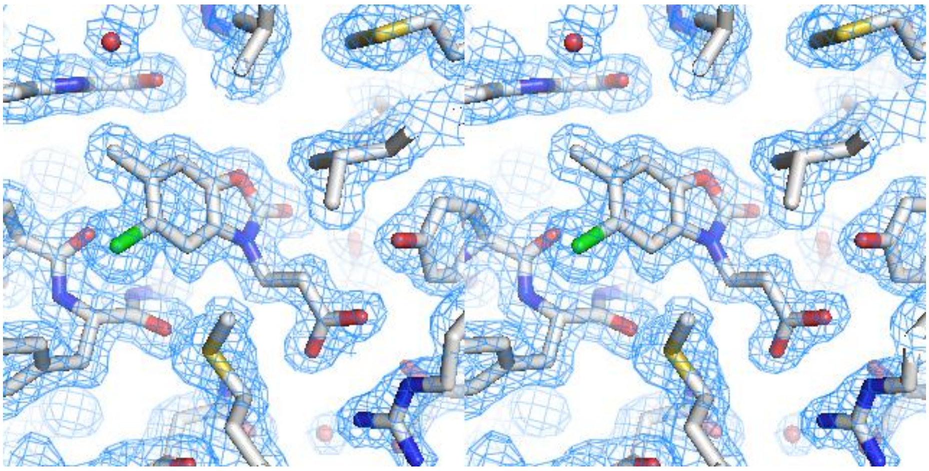

GSK775(B2) complex (A subunit)

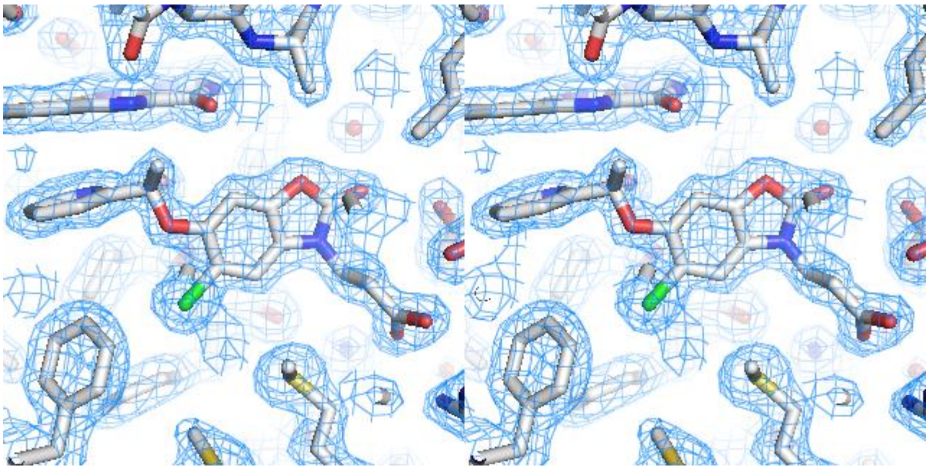

Final $2F_o$-F_c electron density maps contoured at 1[illegible] (blue) superposed on the coordinates of the corresponding fully refined crystal structure.

Supplementary Figure 8 cont. Stereoimages of ligand density.

GSK065(C1) complex (A subunit)

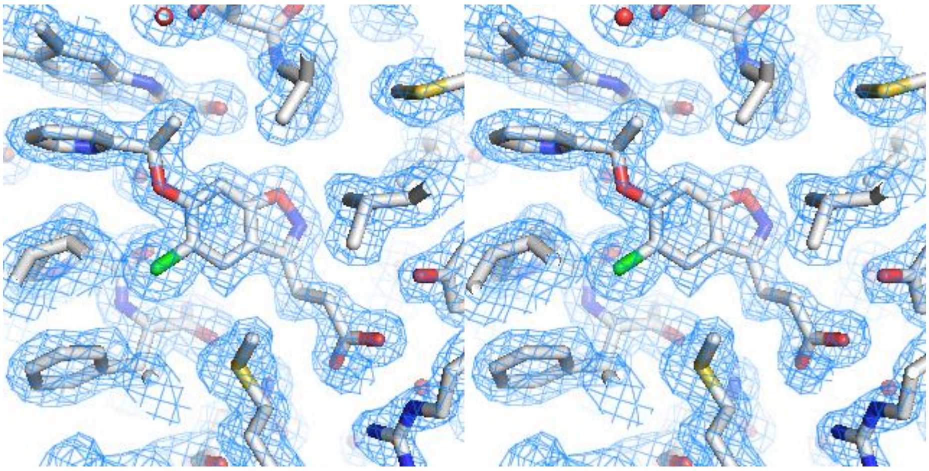

GSK366(C2) complex (A subunit)

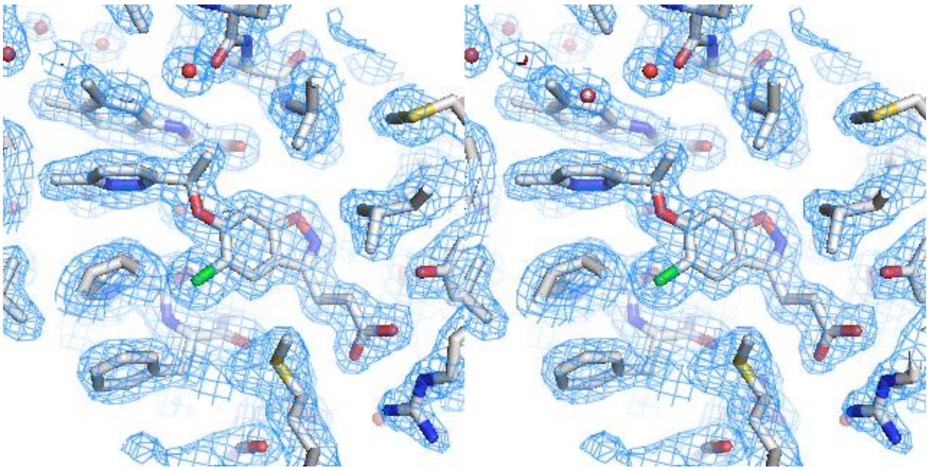

Final $2F_o$-F_c electron density maps contoured at 1[illegible] (blue) superposed on the coordinates of the corresponding fully refined crystal structure.

Supplementary Figure 8 cont. Stereoimages of ligand density.

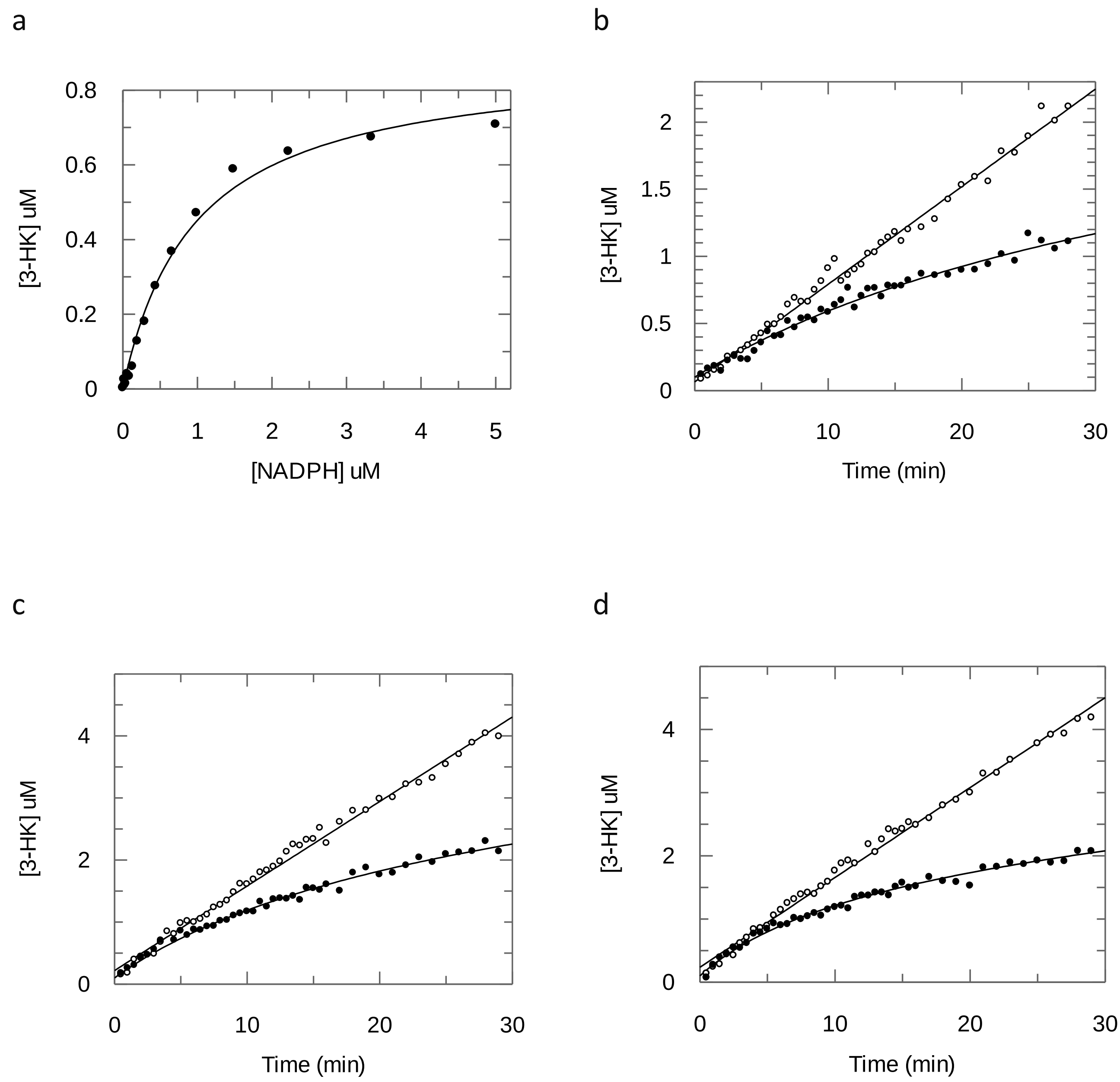

Supplementary Figure 9. Binding kinetics of GSK775(B2) at sub-saturating [NADPH]

(a) Determination of K_M for NADPH against human KMO, at 10 μM Kynurenine. An NADPH regeneration system was used as described in Lowe et al. (2014)[1]. Data were fitted to the Michaelis-Menten equation with K_M = 1.0 μM. **(b-d)** Onset of inhibition timecourses (closed symbols) measured with 10 nM GSK775(B2) at different concentrations of NADPH. k_{obs} values were obtained by fitting to an equation describing onset of inhibition under non-pseudo first order conditions. The corresponding uninhibited timecourses are shown with open symbols and were fitted to a straight line. **(b)** 0.3 μM NADPH, k_{obs} = 0.013 min^{-1}; **(c)** 1 uM NADPH, k_{obs} = 0.035 min^{-1}; **(d)** 5 μM NADPH, k_{obs} = 0.069 min^{-1}.

SUPPLEMENTARY TABLES

Supplementary Table 1. X-ray data collection and refinement statistics.

	Apo	L-kynurenine	GSK428(A2)
Data collection			
Beam line/detector	Diamond IO4-1/Pilatus 6M	Diamond IO4-1/Pilatus 6M	ESRF ID30B/Pilatus 6M
Space Group	$P2_1$	$P2_1$	$P2_1$
Resolution (Å)	134-1.94 (1.98-1.94)	68-1.50 (1.58-1.50)	50-1.62 (1.63-1.62)
Observations	230137 (10056)	482557 (68538)	384501 (3269)
Unique reflections	71621 (3201)	155570 (22560)	116150 (1076)
Redundancy	3.2 (3.1)	3.1 (3.0)	3.3 (3.0)
Completeness (%)	96.4 (86.4)	99.7 (99.6)	95.9 (89.1)
Mean $I/\sigma I$	16.9 (2.5)	8.1 (1.7)	13.7 (1.9)
R_{merge}	0.052 (0.578)	0.062 (0.614)	0.057 (0.603)
$CC_{1/2}$	0.993 (0.542)	0.998 (0.495)	0.998 (0.725)
Refinement			
Resolution (Å)	134-1.94 (1.99-1.94)	68-1.50 (1.54-1.50)	50-1.63 (1.67-1.63)
Reflections	71566 (4680)	155537 (11388)	115718 (7959)
R_{work}/R_{free}	0.201/0.231 (0.280/0.323)	0.189/0.210 (0.246/0.264)	0.166/0.186 (0.246/0.266)
Total number of atoms	7725	8222	8200
Protein atoms	6993	7003	7000
Ligand atoms	120	135	190
Waters	612	1084	1010
Average B-factors ($Å^2$)			
Protein (main chain/side chain)	29.6/38.0	24.1/30.8	21.9/29.2
Ligand atoms	19.7	18.3	21.9
Waters	38.5	41.0	40.1
RMS deviations from ideal values			
Bond lengths (Å)	0.010	0.010	0.010
Bond angles (°)	0.96	0.96	0.97

Values for the highest resolution shell are given in parentheses

Supplementary Table 1 cont. X-ray data collection and refinement statistics.

	GSK775(B2)	GSK065(C1)	GSK366(C2)
Data collection			
Beam line/detector	ESRF ID30B/Pilatus 6M	ID23-1/Pilatus 6M	ID30A-1/Pilatus 2M
Space Group	$P2_1$	$P2_1$	$P2_1$
Resolution (Å)	67-1.76 (1.77-1.76)	50-1.68 (1.74-1.68)	49-1.75 (1.81-1.75)
Observations	305615 (3236)	362111 (27281)	328445 (30375)
Unique reflections	92637 (964)	108786 (10114)	95918 (9341)
Redundancy	3.3 (3.4)	3.3 (2.7)	3.4 (3.3)
Completeness (%)	98.0 (96.0)	99.1 (94.5)	99.8 (99.7)
Mean $I/\sigma I$	12.6 (2.0)	12.9 (1.6)	13.4 (1.5)
R_{merge}	0.057 (0.713)	0.052 (0.634)	0.051 (0.772)
$CC_{1/2}$	0.998 (0.670)	0.999 (0.621)	0.999 (0.611)
Refinement			
Resolution (Å)	67-1.76 (1.81-1.76)	50-1.68 (1.72-1.68)	45-1.75 (1.79-1.75)
Reflections	92615 (6545)	108768 (7465)	95896 (7054)
R_{work}/R_{free}	0.177/0.206 (0.216/0.237)	0.169/0.198 (0.267/0.289)	0.176/0.203 (0.233/0.260)
Total number of atoms	7985	8078	7989
Protein atoms	6972	7008	6991
Ligand atoms	156	154	174
Waters	857	916	824
Average B-factors (Å^2)			
Protein (main chain/side chain)	32.0/39.4	25.9/33.7	34.1/42.3
Ligand atoms	26.1	18.7	24.7
Waters	45.0	42.1	48.9
RMS deviations from ideal values			
Bond lengths (Å)	0.010	0.010	0.009
Bond angles (°)	0.98	0.99	0.96

Values for the highest resolution shell are given in parentheses

Supplementary references

1. Lowe, D.M. et al. Lead discovery for human kynurenine 3-monooxygenase by high-throughput RapidFire mass spectrometry. *J Biomol Screen* **19**, 508-15 (2014).
